# Higher circulating testosterone linked to higher CAD risk in men: Mendelian randomisation and survival analyses

**DOI:** 10.1210/clinem/dgaf582

**Published:** 2026-03-17

**Authors:** Emily J. Morbey, Felix R. Day, Adam S. Butterworth, Nicholas J. Wareham, John R.B. Perry, Ken K. Ong

**Affiliations:** 1 MRC Epidemiology Unit, University of Cambridge School of Clinical Medicine, L3 Institute of Metabolic Science, Cambridge Biomedical Campus, Cambridge CB2 0SL, UK; 2 Victor Phillip Dahdaleh Heart and Lung Research Institute, Biomedical Campus, Papworth Road, Trumpington, Cambridge CB2 0BB; 3 Metabolic Research Laboratory, Institute of Metabolic Science, University of Cambridge School of Clinical Medicine, Cambridge CB2 0QQ, UK; 4 Department of Paediatrics, University of Cambridge, Cambridge CB2 0QQ, UK

**Keywords:** Coronary artery disease, Testosterone, Mendelian randomisation, Survival analysis, Testosterone supplementation

## Abstract

**Context:**

Testosterone supplementation is increasingly widespread and has well established beneficial effects on sexual function and metabolic health. However, there remains uncertainty regarding associated cardiovascular risks.

**Objective:**

Human genetics studies demonstrated that ‘Mendelian randomization’ (MR) approaches recapitulate the beneficial effects of testosterone therapy, here we apply this to cardiovascular disease.

**Design:**

We performed a Mendelian randomisation study to assess the causal effect of higher circulating testosterone on coronary artery disease (CAD). We also tested the phenotypic association between measured circulating testosterone and CAD in the cohort of men aged 40-69.

**Patients or Other Participants:**

Testosterone genetic instrument data had been derived from 425,097 European ancestry adults from the UK Biobank study; and CAD from SNP-level summary statistics from 1,165,690 individuals in CARDIoGRAMplusC4D.

**Main Outcome Measure(s):**

CAD as defined in CARDIoGRAMplusC4D was the main outcome. In longitudinal analyses, CAD was defined according to medical records and self-report.

**Results:**

We found that higher genetically-predicted circulating testosterone conferred higher risk of CAD in men (OR: 1.17, 95% CI [1.07-1.27], *P*=3.32×10^-4^). There was no evidence of an effect in women (OR: 1.01, 95% CI [0.94-1.10], *P*=0.73). The genetic association in men appeared to be mediated by higher blood pressure. In longitudinal observational analyses, a directionally-opposite association was observed in men, likely arising due to confounding by Type 2 diabetes and BMI.

**Conclusions:**

These data suggest increased testosterone may increase risk of cardiovascular disease and recommend this safety concern should be a focus in future clinical trials for testosterone supplementation.

## Introduction

Use of Testosterone supplementation is increasing (1). In men, it is an approved treatment for hypogonadism, which typically manifests with fatigue and sexual dysfunction (2). Evidence from randomised controlled trials (RCTs) shows beneficial effects of testosterone supplementation on sexual function, lean mass and muscle strength (3). Low circulating testosterone is also a risk factor for poor metabolic health (4) and there are increasing numbers of prescriptions for testosterone in ‘eugonadal’ men (with normal circulating testosterone) (5). However, questions remain about the long-term impacts of testosterone levels on other clinical outcomes. There is also interest in the health impacts of testosterone supplementation in women, in whom its use is licensed for low sexual desire, and some hormone replacement therapies (HRT) contain testosterone.

Coronary artery disease (CAD) is the leading cause of mortality and loss of Disability Adjusted Life Years globally (6). Observational studies have linked low measured testosterone with elevated CAD risk in men (8), suggesting that supplementation might have cardioprotective benefits (9,10). RCTs have investigated the impact of testosterone supplementation on CAD risk in men with age-related testosterone decline (‘functional hypogonadism’) (11,12), as well as in men with pathological hypogonadism, caused by disorders of the hypothalamic-pituitary-testicular axis (13). RCTs have been underpowered and have reported conflicting findings; some reported no difference or decreases in cardiovascular events in hypogonadal men treated with testosterone (14), or improved CAD risk markers (15), while others reported increases in intermediate risk factors (12), and cardiovascular events (16), particularly in men with predisposing risk factors, including particular genetic profiles (17). Furthermore, cardiovascular warnings arose from trials where CAD was a secondary outcome (18). In women, some observational studies of testosterone replacement therapy have identified elevated CAD risk (19), while other studies reported no association (20). RCTs of HRTs containing testosterone found no impact on cardiovascular events in women (21). However, few RCTs of testosterone supplementation in men or women measured cardiovascular disease endpoints as primary outcomes, and those that did were underpowered (22).

Mendelian randomisation (MR) uses genetic variants as instrumental variables to infer causal links between exposures and disease (23). MR has been described as analogous to a large RCT, benefitting from long follow-up and large sample sizes by leveraging the data from very large observational studies. Previous MR evidence shows that higher circulating testosterone levels in men appear protective against Type 2 diabetes but increase the risk of prostate cancer (4). Due to the previously reported distinct genetic architectures of testosterone between sexes (4) and the strong possibility of sex-specific effects of testosterone, we used sex-specific genetic instruments for circulating testosterone (4) as well as sex-specific data on the outcome, CAD, and conducted analyses in men and women separately. To distinguish between effects of testosterone and sex hormone binding globulin (SHBG), we use genetic instruments stratified by their effects on total testosterone, free testosterone and SHBG (4). The use of sex and hormone specific genetic instruments, which have been validated with positive control outcomes (4), allows this study to evaluate cardiovascular safety in the absence of sufficiently powered RCT evidence.

## Methods

Two-sample Mendelian randomisation was used to assess the likely causal effects of testosterone and SHBG on CAD, and also to identify possible mediators for any association.

### Data

Genetic instruments for testosterone and SHBG in men and women were comprised of reported variants identified in previous sex-stratified genome-wide association (GWAS) analyses of circulating total testosterone, free testosterone, and SHBG in UK Biobank (4). As previously reported, the arising GWAS signals were categorised by cluster analyses with the same hormone traits into one of four genetic instruments: 1 & 2) male or female testosterone-specific (variants associated with higher circulating total and free testosterone but not with SHBG), and 3 & 4) male or female SHBG (variants with primary effects on higher circulating SHBG and with secondary diverging effects on higher total and lower free testosterone) (Supplementary Tables S1-S4 (25)). Testosterone variants in men were weighted by their effects on free testosterone, and in women by their effects on total testosterone, since these traits showed the most consistent associations in each cluster (4). Sex-specific summary level data for variant associations with CAD were obtained from a reported meta-analysis of the CARDIoGRAMplusC4D genetics consortium (Coronary ARtery DIsease Genome wide Replication and Meta-analysis (CARDIoGRAM) plus the Coronary Artery Disease (C4D)) and also UK Biobank (26,27). Lipid levels and blood pressure were considered as potential mediators in genetic association models. For these, we obtained summary level, sex-specific variant associations with HDL-cholesterol, LDL-cholesterol, and triglycerides from the Global Lipids Genetics Consortium (28) and for diastolic and systolic blood pressure measured in mmHg, from a sex-specific GWAS in UK Biobank (29) (Supplementary Table S9 (25)).

Phenotypic data on incident CAD in UK Biobank men were derived from hospital electronic records, self-reported conditions and operations at baseline, and death records. CAD was defined using ICD10 codes I21-I23, I24.1, I25.2; ICD-9 codes 410-412, 429.79, self-report of heart attack or myocardial infarction (UKB code 1075 in field 20002; code 1 in field 6150); or self-report of coronary angioplasty or coronary artery bypass grafts (OPCS-4 codes K40.1-4, K41.14, K45.1-5, K49.1-2, K49.8-9, K50.2, K75.1-4, K75.8-9; UKB codes 1070, 1095 in field 20004). This definition of CAD in UK Biobank has been described elsewhere (30). Individuals who had CAD at or prior to recruitment were excluded from phenotypic analyses.

We assessed QRISK®3 cardiovascular risk factors (31) as potential mediators or confounders in phenotypic association models. QRISK includes information on age, ethnicity, deprivation, systolic blood pressure (SBP), SBP variability, body mass index (BMI), total cholesterol to high-density lipoprotein (HDL) cholesterol ratio, smoking status, family history of coronary heart disease and medical history of various diseases. We identified data on these QRISK®3 variables in UK Biobank (Box 1. Supplementary Table S13 (25)). To assess variability in SBP, we calculated the standard deviation of the first 2 SBP measures after recruitment in each individual. All other QRISK®3 variables were based on the information recorded at baseline. Information on age of diagnosis of coronary heart disease in family members was unavailable in UK Biobank, so all familial cases were assigned to ‘diagnosis before age 60’.

### Statistical analysis

The genetic instruments for male testosterone, female testosterone male SHBG, and female SHBG were used as the exposure in separate MR models. Where a variant was not present in the outcome GWAS CAD dataset, we identified a proxy with the highest r^2^ value (all r^2^>0.7) calculated using a random sub-sample of the UK Biobank white British sample, n=20,000. Including proxies, the genetic instruments comprised 95 SNPs for male testosterone, 220 SNPs for female testosterone, 306 SNPs for male SHBG and 322 SNPs for female SHBG (Supplementary Tables S5-S8 (25)). Genotypes at all variants were aligned to the hormone-increasing allele.

The inverse-variance-weighted (IVW) MR model was selected as the primary model if the MR-Egger intercept indicated absence of horizontal pleiotropy (*P_intercept_*>=0.05). If horizontal pleiotropy was present (*P_intercept_*<0.05), we selected the MR-Egger model. We performed MR-PRESSO to identify any individual variants with outlier effects, and re-ran models with these removed. We also ran leave-one-out analysis, where we iteratively removed each of the SNPs in our instrument from the MR model to ensure that the causal effect estimate where not being driven be the effects of just one SNP. In order to calculate the required sample size in a randomised controlled trial to detect the odds ratio calculated in the Mendelian randomisation in our study, we performed sample size calculations using the powerMediation package (version 0.3.4.). We assumed a baseline event rate of 7.3% in the male population, based on the incidence of CAD in the control group of the largest RCT of the impact of testosterone therapy on CAD risk (14). We also used a two-sided alpha of 0.05, 90% power and the odds ratio calculated in our Mendelian randomisation analyses.

Potential mediators were included as genetic covariates in separate sex-specific two-sample multivariable MR (MVMR) models (32). Using genetic instruments for male testosterone and female testosterone, we obtained estimates for their associations with systolic blood pressure, diastolic blood pressure, and lipid levels (including LDL, HDL, and triglycerides), as well as their associations with CAD. GWAS summary statistics were harmonized across the exposure (testosterone), the potential mediators (blood pressure and lipids), and the outcome (CAD), ensuring alignment of effect alleles across datasets. We then applied MVMR to model the impact of testosterone on CAD while adjusting for the genetic associations of testosterone on lipid levels and blood pressure (32).

Cox proportional hazards models were used to test the phenotypic association between measured circulating testosterone and CAD in the cohort of men aged 40-69 at baseline in UK Biobank (N=228,989), with time since recruitment as the underlying time variable. Models compared men with measured testosterone <12 nmol/l to those with higher levels, as this threshold is typically used in clinical practice to indicate low testosterone (33). As covariates, QRISK®3 variables were included altogether and individually in separate models.

We performed analyses using the MendelianRandomization (34), Survival (35), and MRPRESSO (36) packages in R version 4.3.2. software platform (R Development Core Team, Vienna, Austria).

## Results

### Sex-specific Mendelian randomisation of sex hormones with coronary artery disease

We analysed sex-specific genetic variant data on 425,097 individuals for sex hormone levels and 1,165,690 individuals for CAD, including 181,522 cases. In our initial IVW model, higher genetically-predicted testosterone conferred higher risk of CAD in men (OR: 1.10, 95% CI [1.01-1.19], I^2^=41.2%, *P*=0.03) (Supplementary Table S10 (25)). However, an outlier SNP (rs56196860) was detected by MR-PRESSO (Supplementary Table S11 (25)). rs56196860 is a missense variant in *FKBP4*, which is a reported candidate gene for androgen insensitivity syndrome (37). Leave-one-out analysis indicated this model underestimated the true effect of circulating testosterone on CAD due to the presence of this outlier SNP, as the beta estimate in the IVW model was elevated once this SNP was excluded (Supplementary Figure 2 (38)). After excluding this, higher genetically-predicted testosterone conferred a clearer higher risk of CAD in men; each SD higher testosterone conferred a 17% higher risk of CAD (OR: 1.17, 95% CI [1.08-1.27], I^2^=26.9, *P*=3.32×10^-4^). This finding remained significant with a Bonferroni adjustment based on four tests (*P*<0.0125). Similar findings were observed in sensitivity MR models (MR Egger and MR Median) (Table 1). We used the powerMediation package (v0.3.4) in R to estimate the sample size needed to detect an odds ratio of 1.17 in a randomized controlled trial. Assuming 90% power, a 7.3% baseline incidence for CAD (which was the incidence in the control arm of a recent trial of testosterone supplementation (14), and a two-sided alpha of 0.05 the required sample size was 6,439.

Regarding potential mediators, genetically-predicted testosterone conferred higher diastolic BP (*β*=0.050, 95% CI [0.02-0.08], *P*=0.001) and systolic BP (*β*=0.03, 95% CI [0.004-0.063], *P*=0.024), measured in mmHg (Supplementary Table S12 (25)). Neither HDL cholesterol, LDL cholesterol nor triglyceride levels showed significant associations with testosterone in Mendelian randomisation analyses (HDL-c: *β*=-0.02, 95% CI [-0.06-0.001], *P*=0.23, LDL-c: *β*=-0.01, 95% CI [-0.03-0.01], *P*=0.33, Triglycerides: *β*=-0.06, 95% CI [-0.03-0.02], *P*=0.68), and so were not taken forward for multivariable MR (MVMR) models. The inclusion of diastolic BP in a two-sample MVMR model attenuated the genetic association between testosterone and CAD in men (CADadjDBP, OR: 1.09, 95% CI [0.88–1.35], *P*=0.43).

By contrast, higher genetically-predicted testosterone had no impact on CAD risk in women in primary (OR: 1.01, 95% CI [0.94-1.10], *P*=0.73) or sensitivity models (Table 1). While there was moderate heterogeneity among individual variant estimates (I^2^ =43.3%), there was no evidence of directional pleiotropy (*P*_intercept_=0.46) and results were consistent across sensitivity MR models (MR Egger and MR Median).

In IVW models, higher genetically-predicted SHBG levels appeared to confer lower risk of CAD in both men and women. However, there was evidence of directional pleiotropy (both *P*_intercept_<0.05), and the MR-Egger estimates were not statistically significant (Table 1). In addition, rs1799941, located within the *SHBG* gene and with a specific effect on SHBG levels, showed no association with CAD in men (OR: 0.99, *P*=0.43) or women (OR: 1.004, *P*=0.77). Taken together, we found no evidence for an effect of SHBG on CAD in men or women.

### Phenotypic analyses in UK Biobank

To assess the risk of incident CAD associated with low testosterone in men, we performed phenotypic analyses of a binary measured testosterone variable. Complete data on measured testosterone levels, all QRISK variables (39), and time-to-event data on CAD were available for 142,149 men in UK Biobank (Supplementary Table 13 (25)). Descriptive statistics relating to relevant QRISK variables are presented in Table 2.

During a median 15 years follow-up of these men (2,097,177 person-years), there were 9862 incident CAD cases. Before adjustment for QRISK covariates, measured testosterone <12 nmol/l at baseline conferred 15% higher hazard of CAD (HR=1.15, 95% CI [1.10-1.20], *P*=8.6×10^-12^). After adjusting for all QRISK variables, this association was fully attenuated (HR=0.97, 95% CI [0.93-1.01], *P*=0.11) (Supplementary Table S14 (25)). The QRISK variables with the largest impact on the phenotypic association were: Type 2 diabetes (54.3% attenuation) and BMI (48.1%) (Supplementary Table S15 (25)). Notably, inclusion of systolic BP as a covariate slightly attenuated the association between low testosterone and CAD in men (adjusted HR=1.11, 95% CI [1.06-1.15], *P*=8.0×10^-7^).

## Discussion

### Principal findings

Using genetic inference, we identified an apparent causal association between higher circulating testosterone levels and higher CAD risk in men, with no association in women. The genetic association in men was consistent across various sensitivity analyses and appeared to be mediated by the apparent effect of testosterone on higher blood pressure, which shows consistency with the effect of testosterone on blood pressure reported in RCTs of testosterone supplementation (11). This association between testosterone and CAD risk in men is consistent with evidence which shows increased coronary plaque volume in men treated with testosterone (12). Conversely, in longitudinal phenotypic analyses, high circulating testosterone levels at baseline appeared to be protective for CAD in men, but this was likely due to confounding by T2D and BMI.

### Comparison with other studies

Previous RCT evidence on the impact of testosterone supplementation on CAD is inconsistent and likely underpowered (12, 14, 15, 16), while observational studies of this topic benefit from larger sample sizes, but carry limitations and the potential for selection bias (40). For example, a population-based matched cohort study gathered a sample size of over 30,000 men, composed of 10,311 men treated with testosterone replacement therapy and 28,029 controls, and found that longer term exposure to testosterone supplementation was associated with reduced risks of mortality, cardiovascular events and prostate cancer (40). Short durations of therapy increased the risk of mortality and cardiovascular events however (40). The largest RCT to date, the TRAVERSE trial involving 5,246 men with low testosterone levels, reported no harmful impact of testosterone supplementation on a composite of major cardiovascular events (hazard ratio, 0.96; 95% confidence interval, 0.78 to 1.17; P<0.001 for non-inferiority) (11). Larger trials to investigate the safety and efficacy of testosterone therapy should be encouraged. To detect the odds ratio of 1.17 as found in our study would require a RCT of over 6,400 men.

Previous MR analyses have produced differing findings. A previous MR study using a much smaller CARDIoGRAMplusC4D dataset of 547,261 individuals including 122,733 CAD cases reported a protective effect of testosterone on risk of CAD and atherosclerotic outcomes in men, apparently mediated by improved lipid profiles (41). However, that study used a sex-combined genetic instrument for testosterone, CAD and sex-combined data for the mediators (41). Our previous work demonstrated the need for sex-stratified MR analyses using sex-specific variants for testosterone due to its complete sex-specific genetic regulation (genetic correction between sexes = 0.001) (4). This suggests there may be multiple pathways in the regulation of testosterone levels, which produce differing impacts on CAD. Another MR study reported a null association between total testosterone and CAD in men, but it used a much smaller outcome sample of 94,478 men including 14,315 cases, and an unrefined (non-cluster derived) testosterone instrument (42). A third, older, MR study found increased risks of thromboembolism, myocardial infarction and heart failure in men, but it used a very limited genetic instrument for testosterone comprising of variants from only two loci (*JMJD1C* and *SHBG*) (43). Variants in *SHBG* increase levels of total testosterone but without altering free or bioactive testosterone (4). Our current and previous findings (4) support the use of clustered, instruments to distinguish between the effects of testosterone and SHBG.

### Meaning of the study

These results are notable as testosterone prescriptions have increased significantly, due to changing prescription guidelines and increased marketing, despite steady rates of pathological hypogonadism in men (1,44). Testosterone replacement therapy for male hypogonadism requires the presence of characteristic signs and symptoms as well as low serum concentrations of total testosterone (<12 nmo/L) or free testosterone (45). These trends have raised concerns about the safety of testosterone supplementation in men, beyond the requirement to monitor for prostate cancer (45). In light of the uncertainty regarding cardiovascular risk associated with testosterone supplementation, Food and Drug Administration guidance issued in 2015 requires health care professionals in the USA to inform patients of this possible risk and that all prescription testosterone products are labelled accordingly (46). By contrast, there is no UK national guideline for testosterone prescribing in men. Our results support the need for more consistent warnings about the possible cardiovascular risks of testosterone supplementation while the RCT evidence is uncertain.

In women, testosterone use has surged, with a 10-fold increase in prescriptions from 2015 to 2022 (47). Testosterone products licenced for men are prescribed off-label to women at lower doses (48), driven by demand to treat low libido and claims of improved brain function and wellbeing. This is concerning given genetic evidence linking higher testosterone in women with higher risks of T2D, endometrial and breast cancers, PCOS and gallstones (4,41). While RCTs in women have identified adverse effects of testosterone on lipid profiles, the impact varied by delivery method (21). Our study found no effect of genetically-predicted testosterone levels on CAD in women, but data on other cardiometabolic outcomes are needed, as well as RCTs featuring cardiovascular conditions as primary outcomes (48).

### Strengths and limitations

This study has several limitations. MR estimates effects of lifelong differences in endogenous testosterone. These may differ from the effects of exogenous testosterone treatment, which might vary by duration, dosage and age. Exogenous testosterone treatment may also exert biphasic effects, with short term risks to health but longer term benefits, which cannot be captured in an MR analysis (40). However, similar studies using this genetic instrument have confirmed the established association between testosterone and risk of prostate cancer (4). Furthermore, MR models poorly estimate non-linear or threshold effects, which are relevant to testosterone as most prescriptions are for men with hypogonadism. MR studies can produce unreliable findings due to inclusion of SNPs with pleiotropic effects. We excluded one SNP (rs56196860) which we identified statistically as an outlier; this decision is supported by its purported role in causing androgen insensitivity syndrome (30), a condition where the functional link between circulating levels of testosterone and its bioaction is broken. While our sensitivity analysis diminished the likelihood of any major bias, we were unable to further validate the testosterone results in a biologically relevant manner as testosterone lacks a cis-SNP, unlike SHBG.

Strengths of this study include the use of clustered instruments to separate SHBG and testosterone effects. We leveraged the most recent GWAS of CAD to optimise the power of our two-sample MR analysis, and used sex-specific GWAS results for CAD to estimate sex-specific effects more precisely. Testosterone prescriptions have also been linked to possible increased risk of stroke (46); however, we were unable to address this due to the lack of openly available sex-specific GWAS data on that outcome.

## Conclusions

Sex-specific genetic causal modelling indicates that higher circulating testosterone increases the risk of CAD in men, mediated by the apparent effect of testosterone on higher blood pressure. While MR measures lifelong exposure to testosterone and not exposure to administered testosterone compounds, these findings support guidance to inform patients of the possible cardiovascular risks of testosterone supplementation while the RCT evidence is uncertain.

## Figures and Tables

**Table 1:** Results of Mendelian randomisation of sex hormone levels to coronary artery disease

	Exposure	SNPs found	Proxies used	Total SNPs	Model	OR	95% CI	*P*	I^2^ (%)
	SHBG	245	61	306	IVW	0.79	(0.70-0.89)	1.22×10^-4^	63.6
					Egger	0.92	(0.77-1.10)	0.38	97.8
					*Intercept*			0.02	
					Median	0.94	(0.82-1.09)	0.40	
**Male**	Testosterone	74	20	94	IVW	1.17	(1.07-1.27)	3.32×10^-4^	26.9
					Egger	1.24	(1.04-1.48)	0.01	92.0
					*Intercept*			0.43	
					Median	1.22	(1.09-1.37)	<0.001	
	SHBG	259	63	322	IVW	0.72	(0.62-0.83)	6.2×10^-6^	76.9
					Egger	0.88	(0.70-1.11)	0.28	96.2
					*Intercept*			0.03	
					Median	1.02	(0.84-1.24)	0.85	
**Female**	Testosterone	182	38	220	IVW	1.01	(0.94-1.10)	0.73	43.3
					Egger	1.06	(0.92-1.22)	0.41	95.7
					*Intercept*			0.46	
					Median	1.08	(0.96-1.22)	0.19	

**Table 2:** Descriptive statistics of testosterone deficient and testosterone sufficient men in UKBB with complete time to event and covariate data

Variable	Testosterone Deficient (<12nmol/L)	Testosterone Sufficient (>=12nmol/L)	*p*
**n**	80,059	67,463	
**Age at recruitment (mean (SD))**	57.37 (7.96)	56.67 (8.19)	<0.001
**CAD (%)**	7.15	6.24	<0.001
**BMI (mean (SD))**	28.80 (4.41)	26.70 (3.69)	<0.001
**Type 2 Diabetes (%)**	16.0	8.1	<0.001
**Hypertension (%)**	27.9	19.1	<0.001
**Corticosteroid use (%)**	0.13	0.12	0.70
**Antipsychotic use (%)**	0.06	0.05	0.64
**Rheumatoid Arthritis (%)**	1.76	1.58	0.008
**Atrial Fibrillation (%)**	12.21	11.25	<0.001
**Kidney Disease (%)**	4.30	2.56	<0.001
**Migraine (%)**	1.46	1.41	0.44
**Systemic Lupus Erythematosus (%)**	0.05	0.07	0.12
**Mental Illness (%)**	2.15	1.83	<0.001
**Erectile Dysfunction (%)**	0.33	0.36	0.44
**Townsend Deprivation Index (mean (SD))**	-1.38 (3.06)	-1.23 (3.15)	<0.001
**Cholesterol (mean (SD))**	5.48 (1.13)	5.59 (1.08)	<0.001
**HDL-c (mean (SD))**	1.24 (0.30)	1.34 (0.32)	<0.001
**Systolic blood pressure (mean (SD))**	142.52 (17.22)	139.84 (17.39)	<0.001
**Systolic blood pressure SD (mean (SD))**	5.38 (4.39)	5.25 (4.32)	<0.001
**Cholesterol:HDL (mean (SD))**	4.59 (1.18)	4.34 (1.12)	<0.001
**Family history of CAD (%)**	40.2	38.4	<0.001
**Ever smoked (%)**	66.2	65.1	<0.001

## Data Availability

This research was conducted using the UK Biobank Resource under application 9905. The data reported in this paper are available to other investigators by application directly to the UK Biobank. The genetic associations with the outcomes in the UK Biobank and CARDIoGRAMplusC4D consortium are provided in the supplementary data. Software code in R for implementing the Mendelian randomisation analysis and survival analysis is available on GitHub at: https://github.com/emilymorbey/BMJ_FINAL_TESTOSTERONE/blob/main/Testosterone_cad_mr.r

## Box 1 Variables used in QRISK3 algorithm

Age at study entry (baseline)

Ethnic origin (nine categories)

Deprivation (as measured by the Townsend score, where higher values indicate higher levels of material deprivation)

Systolic blood pressure

Body mass index

Total cholesterol: high density lipoprotein cholesterol ratio

Smoking status (non-smoker, former smoker, light smoker (1-9/day), moderate smoker (10-19/day), or heavy smoker (≥20/day))

Family history of coronary heart disease in a first degree relative aged less than 60 years

Diabetes (type 1, type 2, or no diabetes)

Treated hypertension (diagnosis of hypertension and treatment with at least one antihypertensive drug)

Rheumatoid arthritis (diagnosis of rheumatoid arthritis, Felty’s syndrome, Caplan’s syndrome, adult onset Still’s disease, or inflammatory polyarthropathy not otherwise specified)

Atrial fibrillation (including atrial fibrillation, atrial flutter, and paroxysmal atrial fibrillation)

Chronic kidney disease (stage 4 or 5) and major chronic renal disease (including nephrotic syndrome, chronic glomerulonephritis, chronic pyelonephritis, renal dialysis, and renal transplant)

Measure of systolic blood pressure variability (standard deviation of repeated measures)

Diagnosis of migraine (including classic migraine, atypical migraine, abdominal migraine, cluster headaches, basilar migraine, hemiplegic migraine, and migraine with or without aura)

Corticosteroid use (British National Formulary (BNF) chapter 6.3.2 including oral or parenteral prednisolone, betamethasone, cortisone, depo-medrone, dexamethasone, deflazacort, efcortesol, hydrocortisone, methylprednisolone, or triamcinolone)

Systemic lupus erythematosus (including diagnosis of SLE, disseminated lupus erythematosus, or Libman-Sacks disease)

Second generation “atypical” antipsychotic use (including amisulpride, aripiprazole, clozapine, lurasidone, olanzapine, paliperidone, quetiapine, risperidone, sertindole, or zotepine)

Diagnosis of severe mental illness (including psychosis, schizophrenia, or bipolar affective disease)

Diagnosis of erectile dysfunction or treatment for erectile dysfunction (BNF chapter 7.4.5 including alprostadil, phosphodiesterase type 5 inhibitors, papaverine, or phentolamine)
